# Women’s and girls’ experiences of reproductive coercion and opportunities for intervention in family planning clinics in Nairobi, Kenya: a qualitative study

**DOI:** 10.1186/s12978-020-00942-7

**Published:** 2020-06-17

**Authors:** Sabrina C. Boyce, Jasmine Uysal, Stephanie M. DeLong, Nicole Carter, Chi-Chi Undie, Wilson Liambila, Seri Wendoh, Jay G. Silverman

**Affiliations:** 1 Center on Gender Equity and Health, School of Medicine, University of California – San Diego, 9500 Gilman Dr., La Jolla, CA 92093 USA; 2 Population Council, Nairobi, Kenya; 3 International Planned Parenthood Federation, London, UK

**Keywords:** Reproductive health, Intimate partner violence, Sub-Saharan Africa, Family planning clinics, Reproductive coercion

## Abstract

**Background:**

Reproductive coercion (RC), which includes contraceptive sabotage and pregnancy coercion, may help explain known associations between intimate partner violence (IPV) and poor reproductive health outcomes, such as unintended pregnancy. In Kenya, where 40% of ever-married women report IPV and 35% of ever-pregnant women report unintended pregnancy, these experiences are pervasive and co-occurring, yet little research exists on RC experiences among women and adolescent girls. This study seeks to qualitatively describe women’s and girls’ experiences of RC in Nairobi, Kenya and opportunities for clinical intervention.

**Methods:**

Qualitative data were collected as part of the formative research for the adaptation of an evidence-based intervention to address reproductive coercion and IPV in clinical family planning counselling and provision in Nairobi, Kenya in April 2017. Focus group discussions (*n* = 4, 30 total participants) and in-depth interviews (*n* = 10) with family planning clients (ages 15–49) were conducted to identify specific forms of reproductive coercion, other partner-specific barriers to successful contraception use, and perceived opportunities for family planning providers to address RC among women and girls seeking family planning services. Additionally, data were collected via semi-structured interviews with family planning providers (*n* = 8) and clinic managers (*n* = 3) from family planning clinics. Data were coded according to structural and emergent themes, summarized, and illustrative quotes were identified to demonstrate sub-themes. Kenyan family planning providers and administrators informed interpretation.

**Results:**

The results of this study identified specific forms of pregnancy coercion and contraceptive sabotage to be common, and often severe, impeding the use of contraceptives among female family planning clients. This study offers important examples of women’s strategies for preventing pregnancy despite experiencing reproductive coercion, as well as opportunities for family planning providers to support clients experiencing reproductive coercion in clinical settings.

**Conclusions:**

Reproductive coercion is a critical barrier to modern contraceptive use in Kenya. Results from this study highlight opportunities for family planning providers to play a critical role in supporting women and girls in their use of contraception when reproductive coercion is present.

## Plain English Summary

Reproductive coercion (RC), which includes male partner behaviors that create barriers to women and girls’ efforts to prevent pregnancy, may help explain known associations between intimate partner violence (IPV) and poor reproductive health outcomes, such as unintended pregnancy. In Kenya, where 40% of ever-married women report IPV and 35% of ever-pregnant women report unintended pregnancy, these experiences are pervasive and co-occurring, yet little research exists on RC experiences among women and adolescent girls. Qualitative data were collected as part of research to inform the adaptation of an intervention to address reproductive coercion and intimate partner violence in clinical family planning services in Nairobi, Kenya in April 2017. Four focus group discussions and 10 interviews with family planning clients (ages 15–49) were conducted. Additionally, data were collected from 8 providers and 3 clinic managers from family planning clinics. Data were organized by themes (developed prior to and during analysis), summarized, and illustrative quotes were identified to demonstrate sub-themes, all of which were then reviewed by local family planning providers to inform interpretation. The results of this study identified specific forms of reproductive coercion to be common and sometimes severe, often impeding the use of contraceptives among female family planning clients. Reproductive coercion is a critical barrier to modern contraceptive use in Kenya. Results from this study highlight opportunities for family planning providers to play a critical role in supporting women and girls in their use of contraception when reproductive coercion is present.

## Introduction

Globally, women who report experiencing intimate partner violence (IPV) are more likely to experience poor reproductive health outcomes, such as unintended pregnancy [1–3]. This issue is particularly relevant in Kenya, where, similar to many low and middle-income countries (LMICs), unintended pregnancy is pervasive, with 10% of all births reported as unwanted and 25% mistimed [4]. Unintended pregnancy is high among women experiencing physical or sexual violence from a male partner, an experience reported by nearly 40% of Kenyan ever-married women ages 15–49 years [4, 5]. IPV may be linked to unintended pregnancy via another form of gender-based violence, reproductive coercion (RC), which has been shown in the United States to be independently associated with risk for unintended pregnancy, above and beyond the risk associated with IPV [6]. Research in many global settings have found that women’s experiences of IPV and RC are positively associated with one another [7–9].

RC is a term that describes specific behaviors enacted by a male partner or family member that directly or indirectly interfere with a woman or girl’s efforts to prevent pregnancy, seek safe abortion, or continue a pregnancy [2]. According to the framework outlined by Miller et al., RC can include pregnancy coercion (i.e., manipulation, threats, or use of violence to pressure a woman to not use family planning and/or get pregnant against her wishes) and contraceptive sabotage (i.e., active interference with use of a contraceptive method by hiding, destroying, or removing it) [10]. Beyond its implications for women’s reproductive autonomy, RC is also associated with increased risk for sexually transmitted infections (STI), post-traumatic stress disorder, and repeat use of emergency contraceptives [6, 11, 12]. A small but growing body of research has begun to document the pervasiveness of this experience globally among women and girls seeking family planning (FP) services [2, 13]. Qualitative research conducted in Sub-Saharan Africa has described male-dominated decision-making as a barrier to women’s FP use [14, 15]. Minimal research to date, however, has investigated the nature of women’s and girls’ experiences of RC or how they cope with this experience in Kenya, or in any other context characterized by a generalized HIV epidemic, where the lack of reproductive autonomy may be particularly harmful and resources may be limited [13].

Programming to assist women and girls experiencing RC and IPV within family planning services is needed, particularly in settings where unintended pregnancy is pervasive. In 2013, the World Health Organization published global guidelines recommending that women and girls experiencing violence be identified and considered by health care personnel [16]. These recommendations build upon those issued earlier by the American College of Obstetrics and Gynecology supporting assessment and counseling of both RC and IPV in family planning (FP) counselling in order to reduce risk for unintended pregnancy [17]. FP clinics have been found to provide a useful setting for one RC intervention, called ARCHES (Addressing Reproductive Coercion in HEalth Settings), in the United States [18]. Currently, however, no known models to effectively address RC and IPV to prevent unintended pregnancy have been identified within clinical services in LMIC settings.

This study seeks to qualitatively describe women’s and girls’ experiences of RC in Nairobi, Kenya and opportunities for intervention in the context of FP clinical services and provision, an essential next step to inform clinic-based and other RC intervention approaches in LMIC.

## Methods

### Study context

Data were collected as part of formative research in preparation for the adaptation of an evidence-based program to address RC and IPV in clinical FP services in Nairobi, Kenya. In 2016, a collaboration across International Planned Parenthood Federation, the national affiliate (Member Association) Family Health Options of Kenya (FHOK), Population Council, and the Center on Gender Equity and Health at the University of California, San Diego, was developed with the aim of adapting an evidence-based intervention, ARCHES, to the context of Kenya. This clinic-based model involves training of existing FP providers to identify and support women and girls experiencing RC and IPV during standard clinic-based counselling interactions, and to empower them with strategies for minimizing their risk of unintended pregnancy (e.g., by utilizing FP methods that are difficult for a male partner to detect or block). To date, this model has been shown in the United States to effectively reduce RC, increase self-efficacy to enact behaviors to enhance autonomy over FP, and increase use and sharing of IPV resources among women and girls in the United States [18, 19]. The adaptation of ARCHES to Kenya is the first adaptation and evaluation of this evidence-based program in an LMIC setting.

### Setting

Nairobi is the 10th largest metropolitan area in the African continent, with 58% of currently married women ages 15–49 using modern contraception [4]. Injectables (24%), pills (13%), and implants (12%) are the most widely used methods [4]. Among ever-married women in Nairobi ages 15–49, 48% reported ever experiencing IPV (45% report experiencing physical violence and 21% sexual violence) from a romantic partner [4].

In Kenya more broadly, those experiencing IPV are no less likely to report using a female-controlled contraceptive (e.g., intrauterine device, pills, injections) than other women, yet they have higher rates of contraceptive discontinuation and of unintended pregnancy [5, 20]. Contraceptive failure and discontinuation, not lack of contraceptive use, may help explain why women experiencing IPV have higher rates of unintended pregnancy. Addressing IPV as a barrier to contraceptive use has been identified and prioritized by FP2020, the set of 2020 FP goals developed from the London Summit on Family Planning 2012 [21], motivating global efforts to recognize this key barrier for women and girls. Kenya, in particular, was designated as a priority country by FP2020, making it critical to address IPV as part of FP services in this setting. As RC is likely the link between IPV and FP use, understanding the role of RC as it contributes to the high rates of unintended pregnancy, as well as context-specific strategies for addressing it, are critical next steps in Kenya.

### Data collection

As a first step to inform the adaptation of the ARCHES model for the Kenyan context, qualitative formative data were collected in April 2017 to explore specific forms of RC, other partner-specific barriers to successful contraception use, and perceived opportunities for FP providers to address RC among women and girls seeking FP services (ages 15–49 years) in Kenya. The adaptation of ARCHES was initiated in four of FHOK’s six clinics in the Nairobi greater metropolitan area; those selected were the clinics in this geography with highest client volume and represented a diverse spread of socio-economic communities. We conducted focus group discussions at these four selected FHOK clinics (*n* = 4 groups, 30 total participants), with two FGDs conducted among adolescent girls and young women (aged 15 to 24 years old) and two FGD conducted among adult women (aged 25 to 49 years old). Sampling was limited to four focus groups, one from each clinic, for pragmatic reasons; data collection was driven by formative work to adapt the ARCHES interventions for these four clinics. Participants in focus groups who self-identified as having experienced RC were recruited to participate in in-depth interviews (*n* = 10) to further deepen our understanding of their experiences. Semi-structured interviews with FP providers (*n* = 8) and clinic managers (*n* = 3) from selected FHOK clinics were also conducted to provide greater understanding of provider experiences in supporting women and girls experiencing RC and IPV, and to identify the best opportunities for clinical intervention.

#### Recruitment and eligibility

Women and girls seeking FP services were recruited for focus groups by local research staff in the waiting rooms of the four FHOK clinics in April 2017. Individuals were privately screened for inclusion (criteria: being female, aged 15–49 years, currently seeking FP services, and ability to provide a mobile phone number at which they could be contacted without compromising their safety). Those who were eligible were asked for contact information for scheduling and were re-contacted once a focus group had been scheduled for that clinic. All contact information was secured on a protected server accessed only by local research staff; all contact information was destroyed immediately after completion of data collection. After participating in the FGD, eligible participants were invited to participate in an individual interview. Family planning providers and clinic managers at selected FHOK clinics were invited to voluntarily participate in an interview. They were instructed to contact research staff to express interest in participating.

#### Process

Focus group and interview instruments were developed collaboratively by US-based and Kenya-based members of the research team and included open-ended questions about experiences of RC and ways in which individuals have responded to these experiences. Data were collected by two Kenyan interviewers who had graduate-level training in research and interviewing, vast data collection experience on various gender-based violence studies, and who were supervised by a regional expert in gender-based violence, as well as qualitative research, from a prominent research institution. Interviews and FGDs were conducted in Kiswahili, audio-taped, and transcribed, including simultaneous translation from Kiswahili to English. In the process of transcription, all personal identifiers were removed and replaced with a participant number linked with gender and age group of the participant. Upon completion of transcription, all audio files were destroyed.

### Ethics

Voluntary signed informed consent was provided before all data collection activities. Participants under age 18 were determined by Kenyan and US ethics boards to be able to provide their own consent due to local laws protecting the confidentiality of youth in accessing family planning services and intimate partner violence services. All study tools and protocols were approved by the University of California, San Diego Institutional Review Board, the Kenyatta National Hospital-University of Nairobi Ethics and Research Committee, and the Population Council Institutional Review Board.

### Analysis

We approached this qualitative research from both a phenomenology and grounded theory perspective, as we aimed to fully describe the lived experience of reproductive coercion for women and girls seeking FP services in our sample, while also allowing each focus group and interview to build our growing understanding of these experiences. Three analysts from the US-based team with extensive experience studying reproductive coercion and conducting global research, and two analysts from the Kenya-based team with a strong understanding of local culture and history of conducting research on interpersonal violence in this region, participated in the analysis.

Based on the RC framework presented by Miller et al. and the data collection instruments [10], a priori structural themes [10] were identified and included pregnancy coercion, contraceptive sabotage, strategies for coping with RC, and provider behavior that enables/restricts FP. Taking a thematic content analysis approach, we then reviewed and hand-coded translated transcripts using an initial codebook made up of these structural themes. Emergent themes were added to the codebook based on coder reviews, and each transcript was reviewed and coded a second time by at least two analysts. Differences in coding were resolved by the last author.

Coded data were summarized by theme, illustrative quotes representing each theme were selected, and analysts created memos to synthesize their growing understanding of each theme throughout the process. Preliminary findings were presented in-person to the clinical partner, including three FP providers from participating FHOK clinics, to elicit further insight and clarify the interpretation of findings. A Standards for Reporting Qualitative Research checklist was used when writing our report [22].

## Results

RC was a common experience discussed by FP clients. All but one interview participant was married, with two reporting that they were in a polygamous marriage (i.e., had a co-wife), and all had children (range: 1–4). Due to methods of sample selection, it was unsurprising that all interview participants reported using family planning and RC from husbands. As a result of the RC, seven clients said that they used FP (mostly injections) secretly (i.e., covertly), without their husbands’ knowledge. Two of the three FP interview participants who reported being able to use FP without having to hide it from their partners, had co-wives. In the FGDs, girls and younger women (ages 15–24) more frequently reported RC, specifically feeling pressured to have a first child, while older women (ages 25–49) more frequently reported RC related to pressure in having more children or more male children.

### Structural and emergent themes

Structural and emergent themes were identified in the data. These structural themes, included pregnancy coercion, direct contraceptive sabotage, strategies for coping with RC, and provider behavior that enables/restricts FP. Emergent themes, within the structural themes, included physically violent pregnancy coercion, pregnancy coercion by in-laws, indirect contraceptive sabotage, method switching, peer support, provider respects client right to autonomy over family planning decisions, and provider disclosure of clients’ confidential health care information (see Table 1). Results are presented in two sections: 1) Client experiences with RC and 2) Experiences with family planning providers.

**Table 1:** Structural and Emergent Themes related to Reproductive Coercion (RC) and Strategies for Facilitating Family Planning Use in the Context of RC

Structural Themes	Emergent Themes
Pregnancy coercion	Physically violent pregnancy coercion
	Pregnancy Coercion by In-laws
Contraceptive Sabotage - Direct	Contraceptive Sabotage - Indirect
Strategies of Coping with RC	Covert contraceptive use
	Method switching
	Peer support
Provider behavior that restricts family planning	Provider disclosure of clients' confidential health care information
Provider behavior that enables family planning	Provider respects client right to autonomy over family planning decisions

### Client experiences with reproductive coercion

#### Pregnancy coercion

Common across participants’ reported experiences were instances of women being pressured to become pregnant by male partners and in-laws (primarily parents-in-law). Participants described pregnancy coercion as including ongoing and persistent verbal pressure to become pregnant, as well as threats and physical violence as a reaction to women’s attempts to prevent pregnancy. Highlighting this relationship between pregnancy coercion and FP use, one participant shared a story of a woman she knew who was threatened and coerced by her male partner to stop her use of the implant, which was preventing her from becoming pregnant:

*“When the man discovered the contraceptive [implant she was using], he was furious and wanted to know why she was using contraceptives without having borne a child for him. He threatened to beat her up and to pull the implant out. She was confused because she did not know how she could give birth to another child when her first child was still very young. The husband would come home in the evening expecting to find the girl [wife] having removed the implant. This was a point of conflict for the couple since it [the contraceptive implant] is usually clearly visible.”* – Interview, FP Client (married, 2 children, covert FP use)

This story exemplifies how visible forms of FP often made women more vulnerable to RC.

Among married or cohabitating women, some participants described male partners who threatened women with divorce or to leave their partner, threatened to marry a co-wife, have a baby with another woman, or actually withheld money as a means of pressuring women to have more children. One FP client reported her husband’s refusal to let her use FP even after giving birth to two children:

“*I gave birth to one child with him again, but after that he refused, [saying] that I should quit using family planning as I should get another child after a year….He told me that if I am not willing to give birth to another child then he will start looking for alternatives somewhere…to get other women who are willing to sire kids for him….So I have been using family planning…secretly.”* – Interview, FP Client (married, 2 children, covert FP use)

Reflecting this, one of the two interviewed FP clients who shared that they had a co-wife said that her husband married another wife because she refused to have more children and was using FP.

Participants shared stories of unmarried women demonstrating that pregnancy coercion persisted outside married and cohabitating relationships. One woman described her friend’s experience of a boyfriend throwing her clothes out of his house when he learned of the friend’s family planning use.

Another participant described a case in which dating partners ended the relationship over FP use, before they were even married or living together:

“*[A friend of mine] is young, and she is not yet 20 years old, and she was using family planning. She had a boyfriend, so when the boyfriend learnt that she was on family planning, he left her, saying that he cannot date a woman who is using family planning methods.” -* FP Client FGD (ages 25–48)

For this man, being unprotected from pregnancy was a prerequisite for dating.

##### Physically violent pregnancy coercion

Physical violence perpetrated by male partners was commonly reported in the context of discovering that their female partners were using contraception without their knowledge, highlighting the common intersections between RC and IPV.

*“I have a sister- in-law who has three children – two boys and one girl – so, she was satisfied with the number of kids she had, but it’s like, her husband wasn’t. So, she was using family planning secretly. Eventually, he suspected she was using injectables and warned her completely against doing that. It became completely unbearable. It then turned to violence in the house.”* – Interview, FP Client (married, 1 female and 3 male children, covert FP use)

In another example, one provider described a client who came to the clinic after being beaten by her husband because he discovered she was using FP,

*“There was another lady of between 28 – 30 years [old]. She came here with bruises all over the body. When I was attending her I thought she had come because of the assault but she had come for the removal of the family planning. When I asked her why [she had been beaten by her husband], she told me that the husband discovered that she was using an implant and she [had] never disclosed [that] to the husband. Then I asked her why [she] didn’t tell [her] husband. She told me that her husband was against family planning from the start. ‘I have 3 children and I cannot handle any more and he cannot also handle any more [yet] he is insisting that I should not use family planning.’”* -FP Provider

While many women experiencing RC found a way to covertly use FP, women who experienced physically violent RC more often reported discontinuing FP use.

##### Pregnancy coercion by in-laws.

Six of the interview participants reported pregnancy coercion from in-laws, including mothers, sisters, and brothers-in-laws. One provider described an FP client’s experience of abuse from her in-laws, who justified the abuse by implying that the woman was not fulfilling her singular purpose in the family, to bear children. The provider recounted how the client’s mother-in-law had said that she would not have allowed the client to marry her son if she had known the client was not going to provide him children. In some other women’s accounts, in-laws created a hierarchy among co-wives or other female relatives, with higher status being ascribed to those with more children, creating pressure to bear more children. One woman told the story of her friend with three children who is now using FP:

“*But the in-laws have sat down and talked about her [the fact that she has stopped bearing children] and she is even discriminated against when there is a [family event]. Preference is given to those with many children. Even when food is being served, her children are served last as they tell her that they are starting with those who have many [children], as hers are few*.” *-*FP Client FGD (ages 25–48)

Sometimes pressure to become pregnant focused on having more male children, even if the woman had already had a son or other female children. Less commonly, clients described situations in which mothers-in-law threatened violence or were verbally or physically abusive in reaction to the woman’s FP use or her desire to not become pregnant which was sometimes coupled with or intensified by pressures from the male partner.

#### Contraceptive sabotage

Evidence of contraceptive sabotage was prominent in the data from both FP clients and providers. Narratives focused on direct and indirect means of sabotage, described by women in this sample, as being perpetrated exclusively by male partners.

##### Direct contraceptive sabotage

Direct sabotage was described by participants as male partners actively interfering with their FP use by destroying the contraceptive method or by forcefully bringing her back to the clinic to remove, or otherwise end, the use of her FP method. One participant described a male partner who sabotaged the effectiveness of a woman’s birth control pills without her knowledge:

*“I know of one man who put his wife’s pills in hot water to reduce their effectiveness, and she conceived. Later, when she started getting nauseous, her husband told her that she might be pregnant, and disclosed what he did.”* - Interview, FP Client (married, 2 children, covert FP use)

One provider echoed a similar story in which the woman’s attempt to use FP were sabotaged and accompanied by threats of divorce:

*“The man eventually saw the pills and you see he discarded the pills and even burned the [clinic card] and told her never to come to the family planning clinic and threatened her that if he finds out that she came to the clinic he will even divorce her*.” – FP Provider

Direct contraceptive sabotage often intensified over time. Some women indicated that once their contraception method was destroyed or taken away, RC and barriers to her FP use increased, resulting in meaningful consequences to her reproductive outcomes. One participant described becoming pregnant after her husband found birth control pills she had hidden under her mattress and threw them away; she then described increased coercion after the resulting birth.

*“When I gave birth to my first child, it was via a [Cesarean] section. So, when I told my husband that I needed time before having another child, he would take nothing of that kind, so I was forced to get the family planning method that I was using secretly. But when he realized that I was using family planning, he took me to the health facility where I was getting the family planning methods and warned the doctors never to administer the family planning methods to me. … I was picked up by my husband and taken to the hospital for the removal of the implant that I had.”* - FP Client FGD (ages 15–24)

This participant described a partner who disregarded her need to heal after giving birth before becoming pregnant again and created barriers for her to access contraception in the future.

##### Indirect contraceptive sabotage

Indirect forms of contraceptive sabotage also played a key role in preventing women from successfully using contraception. Participants described male partners who got angry with them for using contraception and either insisted or used threats to get them to stop using contraception. Participants also described male partners destroying “client cards”, commonly provided by clinics to help patients track injectable contraception appointments ; without these cards, women no longer knew when to return to the clinic for their next contraception doses.

*“There is a neighbor who was using injectables that last for three months, and there was a time that she left for church, and upon the husband searching the house, he found her clinic card in her suitcase. He tore the clinic card into pieces when we got back from church.”* - Interview, FP Client (married, 2 children, covert FP use)

Such indirect sabotage was described as a reason for inconsistent use of FP.

Some women who described pregnancy coercion and contraceptive sabotage also reported that these indirect behaviors lessened or ceased once they had, in the judgement of male partners or in-laws, a sufficient number of children (e.g., over 5 children) or of male children.

#### Women’s strategies to prevent pregnancy despite RC

In the face of pervasive and sometimes severe experiences of RC, women reported finding ways to manage this coercion and continue their efforts to prevent pregnancy. These methods most often included use of contraception without the knowledge of their partner and/or in-laws. Often in combination with such covert use, women described method-switching and the importance of peer support.

##### Covert contraception use

Women’s convert use of contraception (i.e., seeking FP services and using contraception without their partners’ knowledge) was commonly reported, including among seven interviewed FP clients. Women described visiting the FP clinic during times when their partners were away or combining their clinic visits with some other activity their partners found acceptable (e.g., fetching water, taking out the trash, or taking a child for a clinic visit) in order to hide their seeking FP services.

*“I used to go secretly because I was not ready to get pregnant then. … You wait until your partner is not around, and rush to the nearest clinic, get an injection, and hide the clinic card.”* - Interview, FP Client (married, 2 children, covert FP use)

Women who successfully visited clinics for FP services also described using fake names on clinic cards and calendars to eliminate a partner’s suspicion, or asking their provider to store the clinic card for them so that they did not have to take it home.

Participants also provided accounts of changing the location of where they stored their FP method (e.g., from the home to the office) or their FP clinic reminders (e.g., from cards/calendars to phone). Women chose a variety of inconspicuous hiding places in or around their home for their clinic cards in order to maintain their covert use.

*“There is a friend of mine whom I introduced to family planning methods and she was afraid of her husband getting to know [of it]… [S]he told me that she is the one who cleans her husband’s shoes and so she slots the card in her husband’s insole, so when she cleans the shoe, she checks her clinic return date [laughter]. So she goes to the clinic when her husband has gone to work. When she comes back, she slots it back in the husband’s shoe for him to carry it for her.” -*FP Client FGD (ages 25–48)

*“[The client] told me that she wants the pills. So I told her, fine, the pills are here, [we] will give them to you but how will you handle them because the man is in the house? Won’t he take them and throw them away? She told me no…she told me she will put them [with]…the sweets that are kept in small containers. He doesn’t chew [sweets], [so] she told me that she was going to put them in there [the small containers] and mix them and he will just [think] that they are sweets but only [she] will know that there are family planning pills in there.”* – FP Provider

Other participants described hiding their FP methods in bushes nearby their home, in the coal bag, or at the homes of friends.

##### Method-switching

Some women, upon having their use of contraception discovered, chose to switch from their originally preferred method to another, less detectable method in order to continue their covert use and prevent becoming pregnant. Some respondents, especially younger participants, discussed experiencing real or perceived side effects related to their FP method that became noticeable to their partners, who, in turn, suspected they were using a method. The male partners’ discovery of the side effects, and thus the FP use, is what then led them to switch methods:

*“For me I wanted to do family planning because I already have enough children…and now my husband…completely refused. He told me that during intercourse I was becoming so wet and watery…it is as if he realized I was using the pills…. so he refused that method and started quarreling, it is like he knew, so I just decided personally to change the method to avoid the fights, so I just decided to go for the injection…when he asks me what I am using I say there is nothing I am using, because if I tell him that will be a problem” –* Interview, FP Client (married, 1 female and 3 male children, covert FP use)

Like this client, women reported trying to hide the side effects they were experiencing or repeatedly changing FP methods in order to stop side effects their partners had noticed, as a way of keeping their partners from suspecting their FP use.

##### Peer support

Many female FP clients relied greatly on support, encouragement, and active participation in storing FP from other women in order to achieve their pregnancy prevention goals. Many women were introduced to FP methods by friends who took them to the FP clinic for the first time. Other women reported friends who kept their birth control pills or clinic cards for them so they could continue using FP. One client participant said, *“I am even the one who keeps the pills for [my friend] because she cannot keep the pills in her house”* (Interview, FP client: married, co-wife, 2 children, no covert FP use). For many participants, women’s financial saving clubs (i.e. *chamas*), provided an important resource for information and support around FP use, and emerged as a major theme in the data.

*“My friend started off with pills – the ones that you get in three-month batches – just like I did. But her husband noticed as she used to take home many pills so that she didn’t have to go to the clinic frequently. So her sister-in-law saw her taking the pills, and told her husband to go and check his wife’s hand bag, and that is when the husband threw the pills away, and that is when the* Chama *women told her to stop using the pills and start using injectables, as that’s difficult for anyone to notice.” -*FP Client FGD (ages 25–49)

Many women reported that *Chama* members gave them the courage to use FP for the first time or taught them strategies of how to persist in their use of FP after experiencing RC.

### Experiences with family planning providers

#### Provider behavior that restricts family planning

These stories contrast with ones in which providers did not seek private consultation with female clients and removed or refused FP based on male partner demands, prioritizing male partners’ preferences over those of the clients. These experiences denied female clients of the right to make decisions about their own pregnancy prevention efforts and left them vulnerable to unintended pregnancies.

##### Provider disclosure of client’s confidential health care information

Additionally, clients reported that one of the primary barriers to them seeking FP services or disclosing RC was fear that the providers would disclose their confidential reproductive health information and FP use to their male partners, family members, or other community members, especially if the providers were acquainted with the clients’ families or were from the same communities, as was common within these community-based clinics.

“*You see, we live in the same estate, and when I come to this place [the clinic], [a provider at the clinic] knows me, and chances are that she knows my husband. So when I go to the facility, and [she] provides me with the family planning services, [she] might tell her husband, and then her husband goes and tells my husband that I am on family planning – yet, you were doing these things privately. Maybe my husband does not want [me to use family planning]. You see? This will turn into a fight in the house. That is the reason why you will find someone going as far as Kenyatta [National Hospital] because they don’t want to be spotted*.” – Interview, FP Client (married, co-wife, 2 children, no covert FP use)

Some providers also gave other examples of experiences in which angry male partners came to the clinic demanding to know if the provider gave their female partners a FP method. Sometimes these situations felt unsafe to providers, especially if they were not trained on how to handle these types of situations, and in the context of clinics located in communities marked with high levels of violence. Two providers reported feeling compelled to reveal the clients’ information. One provider describes an experience they had of a husband who had beaten his wife for using FP and then came to the clinic demanding to know if the wife was using FP:

“*I did not deny, I told him [the husband] what I gave her. Of course, she has already told the man [husband] and there is no way you [the provider] can deny it. I told him that I gave an injection that will last for three months but after three months I will not give [it to] her again.”* – FP Provider

Such variation in provider responses to RC may leave FP clients feeling unsure of which providers they can trust to support their covert use of FP.

#### Provider behavior that enables family planning

All interviewed providers were empathetic to the clients experiencing RC and all reported cases of RC among their clients; none reported being empathetic to the perpetrators of RC. About half had responded to clients experiencing RC by prioritizing their right to privacy and choice over FP instead of male partner wishes. Two providers talked of acquiescing to male partners’ requests for information and for removing FP methods, without the wife’s consent. Two others prioritized bringing male partners into the clinic to counsel them about FP in hopes of resolving the conflict between the partners around FP; in some cases this was helpful in convincing partners to allow female use of FP and in others, this led to partners making FP decisions for the wife. All providers reported providing women and girls who were experiencing RC, methods of preventing pregnancy that would be less detectable by their male partners.

##### Provider respects client right to autonomy over family planning decisions

Clients and FP providers shared experiences and observations that illuminated opportunities for FP service providers to offer aid and support to women and girls experiencing RC. These included respecting client rights to autonomy over family planning decisions, assisting her with preventing male partner interference with family planning use, and prioritizing clients’ rights to confidential health care. Women reported greatly appreciating providers who worked with them to help them to use a FP method without their male partners knowing and that this was critical to maintaining control of their FP use. Some providers lent this support by suggesting FP methods that a male partner would be less likely to detect, while others allowed women to keep their clinic cards at the clinic rather than requiring them to take the cards home.

Some providers also described helping clients use FP even in the context of difficult appointments in which the clients’ male partners were present and were insisting that the women not use a FP method or use a method different than what the women wanted. In these instances, providers were able to speak privately with their female clients to ask their preferences, then find a clever way to support the clients’ contraception goals while also appeasing their male partners. Some providers, for example, removed the IUDs to show the male partner the “evidence”, but then also discreetly administered a new IUD or a FP injection to the woman. One client shared her experience:

“*…[My husband] took me to the health facility and instructed the doctor to remove the implant from my arm…. But I had already spoken to the doctor about what I was going through, so the doctor [also] gave me an injection that lasts for 3 months. So my husband thinks that I’m not using contraception, and that’s what I am still using to date.”* – Interview, FP Client,

Both clients and providers identified these types of provider responses as being helpful and respectful of female clients’ autonomy around their contraception decisions.

## Discussion

The results of this study identified that experiences of RC were common among this sample of providers and clients seeking FP from community-based clinics in Nairobi, Kenya. Additionally, these results highlight the specific forms of RC women and girls report experiencing, the consequences and severity of RC in promoting unintended pregnancy, and the strategies women and girls are using to prevent pregnancy despite experiencing RC. Consistent with previous research in the United States [10], participants reported forms of RC within the structural themes of pregnancy coercion and contraceptive sabotage. Within each of these themes, subthemes around physical violence, involvement of in-laws, indirect forms of sabotage emerged, offering unique examples of RC to this context and describing a clear link between RC and unintended pregnancy. Consistent with research from other LMIC contexts [13, 23], descriptions of RC from this study highlight the roles of both male partners and in-laws in perpetrating RC. This research uniquely builds upon the current body of qualitative research in Kenya around female decision-making by highlighting RC as a factor inhibiting FP use [24].

This study offers important examples of women’s strategies for preventing pregnancy while experiencing RC, particularly related to covert use of contraception, including switching to less detectable methods, and opportunities for peer and provider support. In participants’ stories, it was common for women to support one another so that they could find a way to store and use FP methods without their partner knowing. Chamas, or women’s micro-finance groups that are common throughout Kenya, emerged as an important source of information and support around RC, and offer an important social system to consider in developing community-based models for RC harm reduction or intervention. Participants’ narratives clarify that many Chamas have been the source of social support to use FP and of strategies to covertly use FP, suggesting that an informal model for how such an intervention might work, already exists. Women’s micro-finance groups have been found to be useful mechanisms for health promotion, including reducing IPV in Sub-Saharan Africa and increasing FP use in other LMIC contexts, suggesting potential utility for addressing RC [25–27].

Results from this current study highlight the need for interventions to prevent and reduce RC, both by shifting male and in-law beliefs and behaviors that directly lead to RC perpetration and by supporting girls and women experiencing RC through routine family planning services so they can access FP resources. Participants’ narratives support the utility of FP clinics as settings for addressing RC, and FP providers as critical allies in supporting women’s reproductive empowerment. However, findings also reinforce the importance of provider training to clarify common biases that prevent providers from prioritizing clients' right to make decisions about their FP use. Additionally, results highlight the need to enact policies and protocols to protect client confidentiality and support the right to use contraception, even when providers are faced with intimidation by male partners. This may be especially true in settings where policies around medical confidentiality have either not been successfully implemented or are poorly enforced, or providers feel more vulnerable to violence. More qualitative and quantitative research is needed to understand how to best prevent male and in-law perpetration of RC, as no known interventions to directly prevent RC exist.

The results of this qualitative study are not without limitations. Typical of qualitative research, these results are not representative of FP clients across Nairobi or Kenya, yet provide an initial window into women’s and girls’ experiences and needs around RC, and directions for clinical intervention. As qualitative data, these results are not an exhaustive or comprehensive listing of forms of RC in this context nor of women’s and girls’ strategies to prevent pregnancy when facing male partner opposition to contraceptives. In particular, RC related to abortion was not included in this study due to political and safety concerns, so future research is needed on this topic in Kenya. Additionally, to help protect the safety of participants, we excluded from the sample those who did not have a safe mobile phone number they could share, which may have led to the exclusion of women most vulnerable to IPV and RC.

The current findings, in combination with a small number of other studies in other LMIC contexts [13], identify the critical need for addressing RC, and informs how this may be best approached in clinical settings. Quantitative research with representative samples of women and girls from such contexts is needed to understand the prevalence of RC, and the socio-demographic, interpersonal and contextual factors associated with this understudied form of gender-based violence. Additionally, efforts are needed regarding integration of these findings into adaptations of clinic-based programs found to effectively address RC is high-income countries [18, 19], and the efficacy of such programs to addressing RC in FP clinics in Nairobi and similar settings.

## Conclusions

Reproductive coercion is a critical barrier to modern FP use in Kenya. The findings from this study on RC in Kenya contribute to a small collection of qualitative evidence from LMIC contexts describing women’s experiences of RC, strategies for preventing pregnancy in the context of RC, and opportunities for support from FP providers. Results from this study highlight opportunities for family planning providers to play a critical role in supporting women and girls in their use of contraception when reproductive coercion is present. More research with representative samples of women and men is needed to build our understanding of risk factors, prevalence, and consequences of RC in this and other LMIC settings.

## Data Availability

The datasets generated and/or analyzed during the current study are not publicly available owing to the duty to protect participant confidentiality as outlined in the informed consent protocol. Versions of the transcripts that have been modified to protect confidentiality are available from the corresponding author on reasonable request.

## Abbreviations

ARCHES: Addressing Reproductive Coercion in HEalth Settings
FHOK: Family Health Options of Kenya
FP: Family planning
IPV: Intimate partner violence
LMIC: Low and middle-income countries
RC: Reproductive coercion
